# Substrate cross-feeding affects the speed and trajectory of molecular evolution within a synthetic microbial assemblage

**DOI:** 10.1186/s12862-019-1458-4

**Published:** 2019-06-20

**Authors:** Elin E. Lilja, David R. Johnson

**Affiliations:** 1 Department of Environmental Systems Science, ETH Zürich, 8092 Zürich, Switzerland; 2 Department of Environmental Microbiology, Eawag, Überlandstrasse 133, 8600 Dübendorf, Switzerland; 3 Present address: School of Physics and Astronomy, University of Edinburgh, Edinburgh, EH9 3FD UK

**Keywords:** Experimental evolution, Cross-feeding, Microbial interactions, Denitrification, Molecular evolution, Mutualism

## Abstract

**Background:**

Substrate cross-feeding occurs when one organism partially consumes a primary substrate into one or more metabolites while other organisms then consume the metabolites. While pervasive within microbial communities, our knowledge about the effects of substrate cross-feeding on microbial evolution remains limited. To address this knowledge gap, we experimentally evolved isogenic nitrite (NO_2_^−^) cross-feeding microbial strains together for 700 generations, identified genetic changes that were acquired over the evolution experiment, and compared the results with an isogenic completely denitrifying strain that was evolved alone for 700 generations. We further investigated how the magnitude of interdependence between the nitrite cross-feeding strains affects the main outcomes. Our main objective was to quantify how substrate cross-feeding and the magnitude of interdependence affect the speed and trajectory of molecular evolution.

**Results:**

We found that each nitrite (NO_2_^−^) cross-feeding strain acquired fewer genetic changes than did the completely denitrifying strain. In contrast, pairs of nitrite cross-feeding strains together acquired more genetic changes than did the completely denitrifying strain. Moreover, nitrite cross-feeding promoted population diversification, as pairs of nitrite cross-feeding strains acquired a more varied set of genetic changes than did the completely denitrifying strain. These outcomes likely occurred because nitrite cross-feeding enabled the co-existence of two distinct microbial strains, thus increasing the amount of genetic variation for selection to act upon. Finally, the nitrite cross-feeding strains acquired different types of genetic changes than did the completely denitrifying strain, indicating that nitrite cross-feeding modulates the trajectory of molecular evolution.

**Conclusions:**

Our results demonstrate that substrate cross-feeding can affect both the speed and trajectory of molecular evolution within microbial populations. Substrate cross-feeding can therefore have potentially important effects on the life histories of microorganisms.

**Electronic supplementary material:**

The online version of this article (10.1186/s12862-019-1458-4) contains supplementary material, which is available to authorized users.

## Background

Substrate cross-feeding is pervasive within microbial communities [1–5]. Substrate cross-feeding occurs when one microbial strain partially consumes a primary substrate into one or more metabolites while other strains then consume the metabolites [2, 6–12]. Substrate cross-feeding is an important feature of many major biogeochemical cycles [3, 4] and may have important effects on the life histories of many microorganisms within the natural environment. However, there are few experimental investigations on how substrate cross-feeding itself influences molecular evolution within microbial populations (e.g. see [13, 14]).

Substrate cross-feeding could increase the speed of molecular evolution because cross-feeding strains must coexist with and can adapt to the traits of partner strains. This may increase the amount of genetic variation for selection to act upon, consequently increasing the speed of molecular evolution. For example, if one strain changes its output of the cross-fed metabolite, then this might stimulate the growth of other strains, increase the supply rate of new genetic changes, and consequently increase the amount of genetic variation for selection to act upon. Additionally, even if the substrate cross-feeding interaction is obligate in nature (i.e., if each substrate cross-feeding strain cannot grow in the absence of the other), there could be competitive interactions for other shared resources at the same time, which could further increase the amount of genetic variation for selection to act upon [15]. Finally, substrate cross-feeding could promote the spatial co-localization of different cross-feeding strains [16, 17], which could in turn promote the loss of different biological functions from each strain over evolutionary time and create even stronger interdependencies [3].

Alternatively, substrate cross-feeding could decrease the speed of molecular evolution. Substrate cross-feeding might result in slower growth rates, which would decrease the supply rate of new genetic changes and consequently reduce the amount of genetic variation for selection to act upon [18, 19]. Substrate cross-feeding could also set the stage for the loss of additional functions and the emergence of more obligate interactions [3]. This would again reduce the amount of genetic variation for selection to act upon. Moreover, the growth of one cross-feeding strain may constrain the growth of the other cross-feeding strain. This could reduce the population density of the other cross-feeding strain, and thus reduce its supply rate of new genetic changes and decrease its speed of molecular evolution [18, 19].

Our main objective was to quantify the effects of substrate cross-feeding between different microbial strains on the speed and trajectory of molecular evolution. In this context, we use the term “speed of molecular evolution” to refer to the number of genetic changes that accumulate per generation and not to the number of genetic changes that accumulate per unit time (e.g. [20]). In addition, we investigated how the magnitude of interdependence between the substrate cross-feeding strains affects the speed and trajectory of molecular evolution. We note here that it was not our aim to identify the molecular mechanisms for why substrate cross-feeding affects the speed and trajectory of molecular evolution, as this might be a system-specific outcome. Instead, we sought to test a more general yet poorly investigated question: does substrate cross-feeding itself have important effects on molecular evolution?

**Fig. 1:**
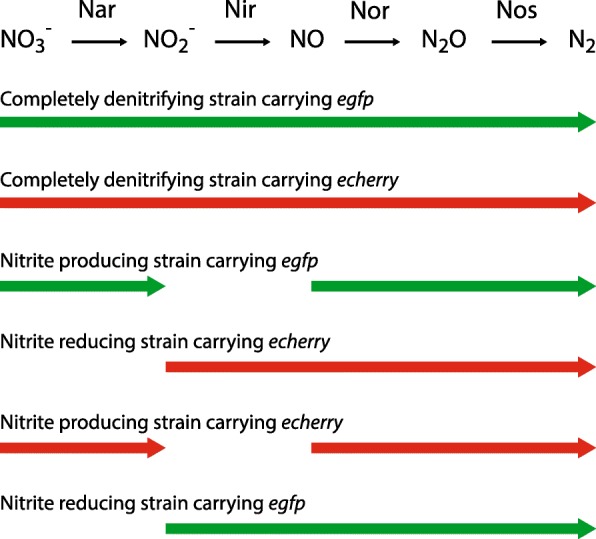
Strains used in this study. Colored arrows indicate the metabolic processes that are performed by each strain. The colors of the arrows indicate whether each strain carries the *egfp* or *echerry* fluorescent protein-encoding gene. Note that we performed the evolution experiment for the nitrite (NO_2_^−^) cross-feeding strains with both combinations of fluorescent proteins (nitrite producing strain carrying *egfp* together with the nitrite reducing strain carrying *echerry* and vice versa). This controlled for any confounding factors that might have emerged due to differences in the metabolic costs associated with expressing the different fluorescent protein-encoding genes. Definitions: Nar, nitrate (NO_3_^−^) reductase encoded by the nar gene cluster; Nir, nitrite reductase encoded by the nir gene cluster; Nor, nitric oxide (NO) reductase encoded by the nor gene cluster; Nos, nitrous oxide (N_2_O) reductase encoded by the nos gene cluster

To achieve our objectives, we used an experimental system based on the bacterium *Pseudomonas stutzeri* A1501, which is a facultative denitrifying bacterium with a fully sequenced genome [21, 22]. Wild-type *P. stutzeri* A1501 can use N-oxides as terminal electron acceptors in the absence of oxygen and encodes all the enzymes required to completely reduce nitrate (NO_3_^−^) to nitrite (NO_2_^−^), nitric oxide (NO), nitrous oxide (N_2_O) and finally to nitrogen gas (N_2_) [22–24] (referred to hereafter as the completely denitrifying strain). The denitrification enzymes are encoded on separate operons [21] (the *nar* gene cluster encodes for nitrate reductase*,* the *nir* gene cluster encodes for nitrite reductase, the *nor* gene cluster encodes for nitric oxide reductase and the *nos* gene cluster encodes for nitrous oxide reductase) and genetic engineering techniques are available to inactivate specific steps of the denitrification pathway [12]. We can therefore create different loss-of-function mutant strains and assemble them together such that they cross-feed specific intermediates of the denitrification pathway [12]. For our experimental system, we previously created a nitrite producing strain that contains a loss-of-function mutation in the *nirS* gene and reduces nitrate to nitrite (referred to hereafter as the nitrite producing strain) [12] (Fig. 1 and Additional file 1: Table S1). We also created a nitrite reducing strain that contains a loss-of-function mutation in the *narG* gene and reduces nitrite to nitrogen gas (referred to hereafter as the nitrite reducing strain) [12] (Fig. 1 and Additional file 1: Table S1). We can then assemble the nitrite producing and reducing strains together in co-culture such that they cross-feed nitrite and measure the consequences of the imposed cross-feeding interaction on ecological and evolutionary processes [12, 25, 26] (Fig. 1). Importantly, the nitrite cross-feeding strains differ from the ancestral completely denitrifying strain at only single genetic loci [12] (Additional file 1: Table S1), thus minimizing confounding factors that could emerge when using more distantly related strains.

An important feature of our experimental system is that the cross-fed metabolite nitrite (NO_2_^−^) is conditionally toxic depending on the pH of the growth environment [12, 24, 27–31]. For our strains under our experimental conditions, nitrite has no statistically detectable toxic effects at pH 7.5 but severe toxic effects at pH 6.5, while pH itself has no statistically detectable effects under the same pH range [12]. We can therefore impose weak interdependence between the nitrite cross-feeding strains by growing them together in co-culture at pH 7.5, where the nitrite reducing strain depends on the nitrite producing strain to provide nitrite but the nitrite producing strain does not measurably depend on the nitrite reducing strain to remove nitrite [12]. Alternatively, we can impose strong interdependence between the nitrite cross-feeding strains by growing them together in co-culture at pH 6.5, where the nitrite reducing strain again depends on the nitrite producing strain to provide nitrite while the nitrite producing strain depends on the nitrite reducing strain to remove nitrite and mitigate its toxic effects [12]. We then experimentally evolved the nitrite cross-feeding strains together in serial batch and identified genetic changes that were acquired over time. Finally, we compared the speed and trajectory of molecular evolution for the nitrite cross-feeding strains with that for an otherwise genetically identical completely denitrifying strain. We reported the methods and outcomes of the evolution experiment for the completely denitrifying strain in a previous study [20]. Importantly, we evolved the nitrite cross-feeding strains using the same experimental conditions and analyzed the genomic data using the same analytical procedures as for the completely denitrifying strain [20]. We also experimentally evolved the nitrite cross-feeding strains together and the completely denitrifying strain alone at the same time.

## Results

### Nitrite (NO_2_^−^) cross-feeding decreases the speed of molecular evolution at the strain level, but only when nitrite has strong toxic effects

We first tested whether the nitrite cross-feeding strains acquired different numbers of genetic changes than did the completely denitrifying strain after 700 generations of experimental evolution. The numbers of genetic changes include all types of genetic changes that we were able to identify using our methodology, including synonymous and non-synonymous point mutations, insertions, deletions, and multiplications [17]. To test this, we isolated one nitrite producing and one nitrite reducing clone from each cross-feeding co-culture and quantified the number of genetic changes acquired by each clone. We then compared the numbers of genetic changes that were acquired among these clones with the numbers of genetic changes that were acquired among randomly selected clones from each completely denitrifying culture (one clone per culture), which we reported in a previous study using the same experimental conditions and analytical procedures [20].

**Fig. 2:**
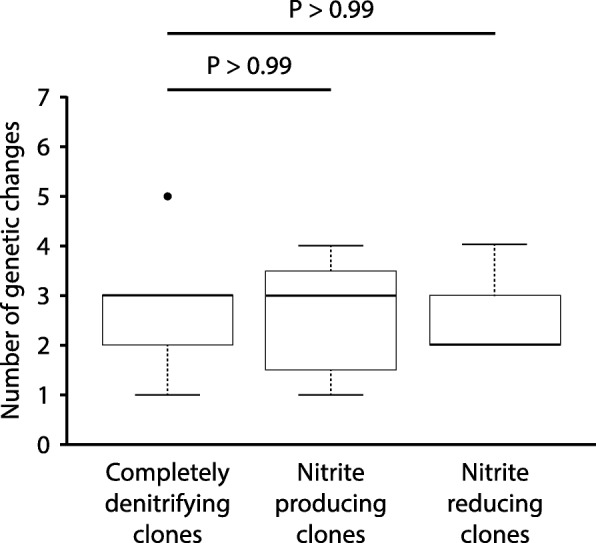
The numbers of genetic changes acquired by clones (one clone per culture) after 700 generations of evolution at pH 7.5 (weak nitrite [NO_2_^−^] toxicity, weak interdependence). The horizontal bars and *P*-values indicate the outcomes from two-sample Wilcoxon rank-sum tests. Data are presented as Tukey box-plots. The numbers of genetic changes include synonymous and non-synonymous point mutations, insertions, deletions, and multiplications. There are no statistical differences in the numbers of acquired genetic changes between the completely denitrifying clones and the nitrite producing or reducing clones

**Fig. 3:**
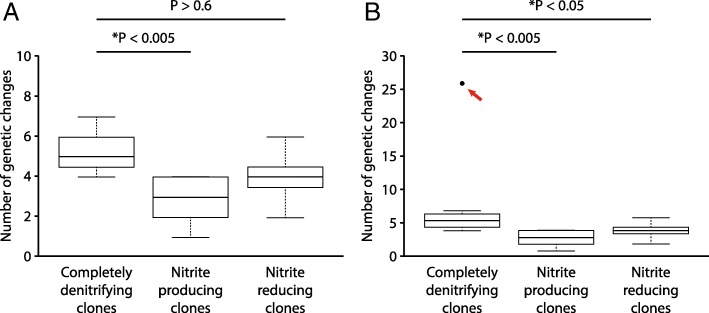
The numbers of genetic changes acquired by clones (one clone per culture) after 700 generations of evolution at pH 6.5 (strong nitrite [NO_2_^−^] toxicity, strong interdependence). The horizontal bars and *P*-values indicate the outcomes from two-sample Wilcoxon rank-sum tests. The stars indicate *P*-values less than 0.05. Data are presented as Tukey box-plots. The numbers of genetic changes include synonymous and non-synonymous point mutations, insertions, deletions, and multiplications. Panels are for **a** data excluding the single completely denitrifying clone with a genetic change in *uvrA*, and **b** data including the single completely denitrifying clone with a genetic change in *uvrA* (indicated by the red arrow). The completely denitrifying clones generally acquired significantly more genetic changes than the nitrite producing or reducing clones

We first performed this comparison for clones evolved at pH 7.5 (weak nitrite [NO_2_^−^] toxicity, weak interdependence). We did not detect any significant differences in the numbers of genetic changes acquired by the nitrite producing or reducing clones when compared to the completely denitrifying clones (Wilcoxon rank-sum test, *P* > 0.99, n_1_ = n_2_ = 8) (Fig. 2). We next performed the same comparison for the clones evolved at pH 6.5 (strong nitrite toxicity, strong interdependence). As reported in our previous study [20], one of the completely denitrifying clones acquired significantly more genetic changes than the other completely denitrifying clones, most likely due to a genetic change in the *uvrA* gene within this single clone [32, 33]. We therefore performed the comparison either including or excluding this clone. When we excluded the single completely denitrifying clone containing the genetic change in *uvrA* from the test, the completely denitrifying clones acquired significantly more genetic changes than the nitrite producing clones (Wilcoxon rank-sum test, *P* < 0.005, n_1_ = 7, n_2_ = 8) but not more than the nitrite reducing clones (Wilcoxon rank-sum test, *P* > 0.6, n_1_ = 7, n_2_ = 8) (Fig. 3a). When we included the single completely denitrifying clone with the genetic change in *uvrA* into the test, the completely denitrifying clones acquired significantly more genetic changes than did either the nitrite producing or the reducing clones (Wilcoxon rank-sum test, *P* < 0.005, n_1_ = n_2_ = 8) (Fig. 3b). Taken together, our data indicate that nitrite cross-feeding decreases the speed of molecular evolution, but only at pH 6.5 when nitrite has strong toxic effects, the magnitude of interdependence is strong, and generation times are slower.

### Nitrite (NO_2_^−^) cross-feeding increases the speed of molecular evolution at the community level

**Fig. 4:**
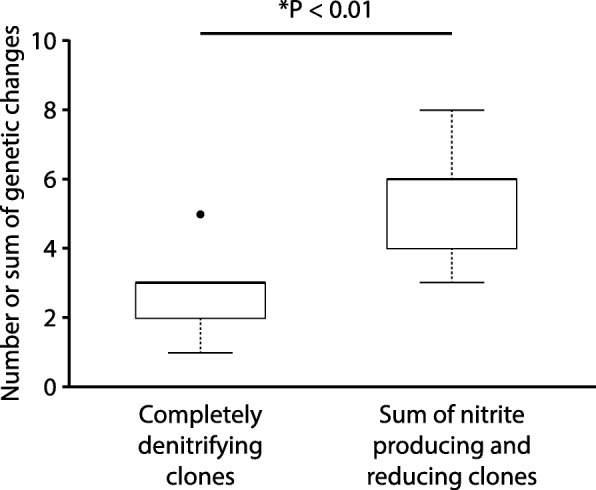
The numbers of genetic changes acquired by the completely denitrifying clones (one clone per culture) compared to the sums of genetic changes acquired by the nitrite (NO_2_^−^) producing and reducing clones (one clone each per culture) after 700 generations of evolution at pH 7.5 (weak nitrite toxicity, weak interdependence). The horizontal bar and *P*-value indicate the outcome from a two-sample Wilcoxon rank-sum test. The star indicates a *P*-value less than 0.05. Data are presented as Tukey box-plots. The numbers of genetic changes include synonymous and non-synonymous point mutations, insertions, deletions, and multiplications. The sums of genetic changes acquired by the nitrite producing and reducing clones are significantly greater than the numbers of genetic changes acquired by the completely denitrifying clones

**Fig. 5:**
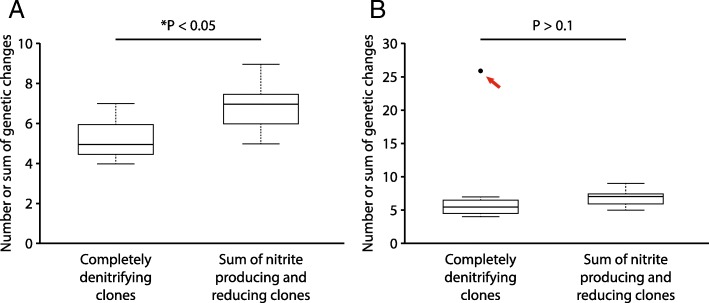
The numbers of genetic changes acquired by the completely denitrifying clones (one clone per culture) compared to the sums of genetic changes acquired by the nitrite (NO_2_^−^) producing and reducing clones (one clone each per culture) after 700 generations of evolution at pH 6.5 (strong nitrite toxicity, strong interdependence). The horizontal bars and *P*-values indicate the outcomes from two-sample Wilcoxon rank-sum tests. The star indicates a *P*-value less than 0.05. Data are presented as Tukey box-plots. The numbers of genetic changes include synonymous and non-synonymous point mutations, insertions, deletions, and multiplications. Panels are for **a** data excluding the clone with a genetic change in *uvrA*, and **b** data including the clone with a genetic change in *uvrA* (indicated by the red arrow). The sums of genetic changes acquired by the nitrite producing and reducing clones are greater than the numbers of genetic changes acquired by the completely denitrifying clones, but only when the clone containing a genetic change in *uvrA* is removed from the analysis

We next compared the numbers of genetic changes acquired by the completely denitrifying clones (one clone per culture) to the sums of genetic changes acquired by the nitrite producing and reducing clones (one clone each per culture). We note here that we never observed a genetic change that was present in both the nitrite producing and reducing clones from a single culture (Additional file 1: Tables S2–S3); the sums of genetic changes therefore do not include any redundant counts. At pH 7.5 (weak nitrite toxicity, weak interdependence), we found that the sums of genetic changes acquired by the nitrite producing and reducing clones was significantly greater than the numbers of genetic changes acquired by the completely denitrifying clones (Wilcoxon rank-sum test, *P* < 0.01, n_1_ = n_2_ = 8) (Fig. 4). At pH 6.5 (strong nitrite toxicity, strong interdependence) this is also true, but only when the clone with the genetic change in *uvrA* is removed from the analysis (Wilcoxon rank-sum test, *P* < 0.05, n_1_ = 7, n_2_ = 8) (Fig. 5). Our results therefore indicate that nitrite cross-feeding does not decrease the speed of molecular evolution at the community level as was observed at the strain level (Figs. 2 and 3), but instead tends to increase the speed of molecular evolution at the community level. This analysis assumes that two randomly selected clones from a single completely denitrifying culture are likely genetically identical (i.e., we assume each completely denitrifying culture is likely dominated by a single genotype at the sampled time point). As a consequence, our analyses are unable to distinguish between genetic changes that became fixed within the cultures and polymorphisms, which would ideally be addressed via population-level re-sequencing.

### Strong nitrite (NO_2_^−^) toxicity increases the speed of molecular evolution for the nitrite reducing clones but not for the nitrite producing clones

**Fig. 6:**
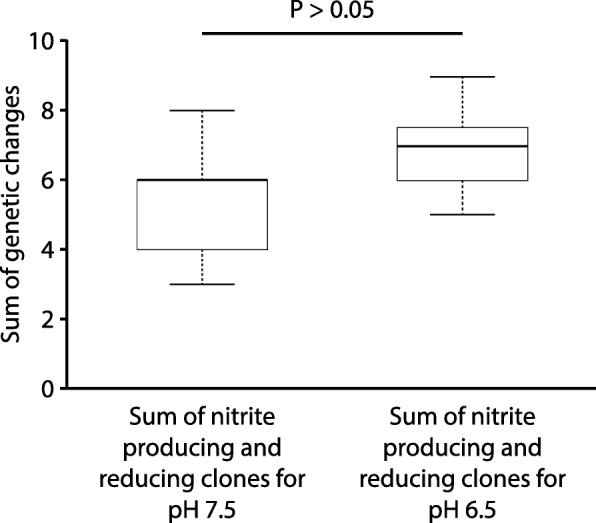
The sums of genetic changes acquired by the nitrite (NO_2_^−^) producing and reducing clones (one clone each per culture) after 700 generations of evolution at pH 7.5 (weak nitrite toxicity, weak interdependence) or pH 6.5 (strong nitrite toxicity, strong interdependence). The horizontal bar and *P*-value indicate the outcome from a two-sample Wilcoxon rank-sum test. The star indicates a *P*-value less than 0.05. Data are presented as Tukey box-plots. The numbers of genetic changes include synonymous and non-synonymous point mutations, insertions, deletions, and multiplications. The sums of genetic changes acquired by the nitrite producing and reducing clones are not significantly different after evolution at pH 7.5 (weak nitrite toxicity, weak interdependence) or pH 6.5 (strong nitrite toxicity, strong interdependence)

**Fig. 7:**
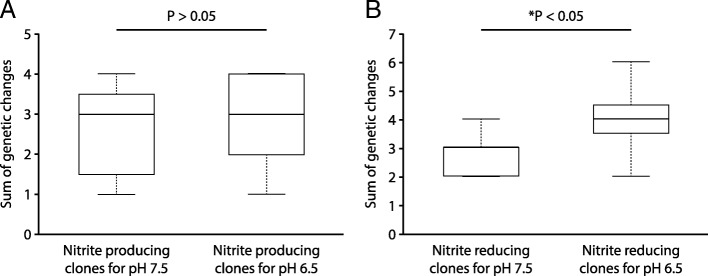
The sums of genetic changes acquired after 700 generations of evolution by **a** nitrite (NO_2_^−^) producing clones (one clone per culture) evolved at pH 7.5 (weak nitrite toxicity, weak interdependence) or pH 6.5 (strong nitrite toxicity, strong interdependence), or **b** nitrite reducing clones (one clone per culture) evolved at pH 7.5 (weak nitrite toxicity, weak interdependence) or pH 6.5 (strong nitrite toxicity, strong interdependence). The horizontal bars and *P*-values indicate the outcomes from two-sample Wilcoxon rank-sum tests. The star indicates a *P*-value less than 0.05. Data are presented as Tukey box-plots. The numbers of genetic changes include synonymous and non-synonymous point mutations, insertions, deletions, and multiplications. The numbers of genetic changes acquired by the nitrite reducing clones are significantly greater after evolution at pH 6.5 (strong nitrite toxicity, strong interdependence) than at pH 7.5 (weak nitrite toxicity, weak interdependence)

We next tested whether the strength of nitrite toxicity (and thus the magnitude of interdependence between the nitrite cross-feeding strains) affects the numbers of genetic changes acquired by the nitrite cross-feeding clones. First, we compared the sums of genetic changes acquired by the nitrite producing and reducing clones (one clone each per culture) evolved at pH 6.5 (strong nitrite toxicity, strong interdependence) with the sums of genetic changes acquired by the nitrite producing and reducing clones (one clone each per culture) evolved at pH 7.5 (weak nitrite toxicity, weak interdependence). We did not detect a significant difference in the numbers of acquired genetic changes (Wilcoxon rank-sum test, *P* > 0.05, n_1_ = n_2_ = 8) (Fig. 6). Next, for the nitrite producing clones (one clone per culture), we compared the numbers of genetic changes acquired after evolution at pH 7.5 (weak nitrite toxicity, weak interdependence) to the numbers of genetic changes acquired after evolution at pH 6.5 (strong nitrite toxicity, strong interdependence). Again, we did not detect any significant difference in the numbers of acquired genetic changes (Wilcoxon rank-sum test, *P* < 0.5, n_1_ = n_2_ = 8) (Fig. 7a). Finally, for the nitrite reducing clones (one clone per culture), we compared the numbers of genetic changes acquired at pH 7.5 (weak nitrite toxicity, weak interdependence) to the numbers of genetic changes acquired at pH 6.5 (strong nitrite toxicity, strong interdependence). In this case, we detected significantly more genetic changes at pH 6.5 (strong nitrite toxicity, strong interdependence) than at pH 7.5 (weak nitrite toxicity, interdependence) (Wilcoxon rank-sum test, *P* < 0.05, n_1_ = n_2_ = 8) (Fig. 7b). Thus, strong nitrite toxicity (and thus stronger interdependence between the nitrite cross-feeding strains) promotes the acquisition of more genetic changes, but only for the nitrite reducing strain that reduces the toxic cross-fed metabolite nitrite.

### Nitrite (NO_2_^−^) cross-feeding affects the trajectory of molecular evolution

**Fig. 8:**
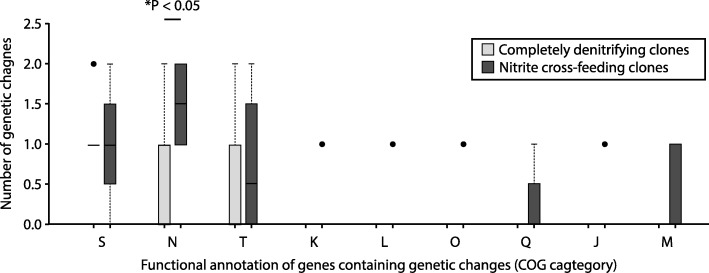
The numbers of genetic changes acquired by clones after 700 generations of evolution at pH 7.5 (weak nitrite [NO_2_^−^] toxicity, weak interdependence) sorted by the functional annotations of the target genes. The horizontal bar and *P*-value indicates a statistically significant outcome from a two-sample Wilcoxon rank-sum test. The star indicates a *P*-value less than 0.05. Data are presented as Tukey box plots. The numbers of genetic changes include synonymous and non-synonymous point mutations, insertions, deletions, and multiplications. The nitrite cross-feeding clones acquired genetic changes that were unique to nitrite cross-feeding. Functional categories (COG): S, function unknown; N, cell motility; T, signal transduction; K, transcription; L, replication, recombination and repair; O, posttranslational modification, protein turnover and chaperones; Q, secondary metabolites biosynthesis, transport and catabolism; J, translation, ribosomal structure and biogenesis; M, cell wall/membrane/envelope biogenesis

We next asked whether the genes containing genetic changes acquired by the nitrite producing and reducing clones have different functional annotations than those acquired by the completely denitrifying clones. Our objective here was to test whether nitrite cross-feeding alters the trajectory of molecular evolution, but not to make statements about the biological consequences of any particular genetic change. To test this, we categorized and compared the genes that acquired genetic changes by their functional annotation [21, 34]. After evolution at pH 7.5 (weak nitrite toxicity, weak interdependence), we found that the nitrite cross-feeding clones and the completely denitrifying clones both acquired genetic changes in genes annotated to cell motility (clusters of orthologous group [COG] N) and signal transduction (COG T) (Fig. 8 and Additional file 1: Table S2). The nitrite cross-feeding clones also sometimes acquired genetic changes in genes annotated to additional functional groups, including genes annotated to secondary metabolite biosynthesis, transport and catabolism (COG Q), and cell wall/membrane/envelope biosynthesis (COG M) (Fig. 8 and Additional file 1: Table S2). A few of these genetic changes occurred multiple times among the nitrite cross-feeding clones but were never detected among the completely denitrifying clones (*PST_3282* and *lpxC*) (Additional file 1: Table S2), suggesting they might be specifically promoted by the nitrite cross-feeding interaction itself.

**Fig. 9:**
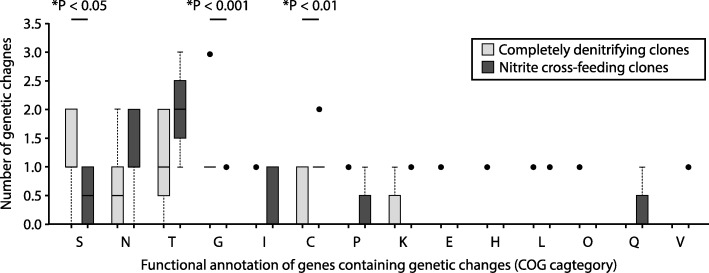
The numbers of genetic changes acquired by clones after 700 generations of evolution at pH 6.5 (strong nitrite [NO_2_^−^] toxicity, strong interdependence) sorted by the functional annotations of the target genes. The horizontal bars and *P*-values indicate statistically significant outcomes from two-sample Wilcoxon rank-sum tests. The stars indicate *P*-values less than 0.05. Data are presented as Tukey box plots. The numbers of genetic changes include synonymous and non-synonymous point mutations, insertions, deletions, and multiplications. Both the nitrite cross-feeding clones and the completely denitrifying clones acquired genetic changes that are unique to nitrite-cross-feeding or complete denitrification. Functional categories (COG): S, function unknown; N, cell motility; T, signal transduction; G, carbohydrate transport and metabolism; I, lipid transport and metabolism; C, energy production and conversion; P, inorganic ion transport and metabolism; K, transcription; E, amino acid transport and metabolism; H, coenzyme transport and metabolism; L, replication, recombination and repair; O, posttranslational modification, protein turnover, and chaperones; Q, secondary metabolites biosynthesis, transport and catabolism; V, defense mechanisms

We next categorized and compared the genes that acquired genetic changes after evolution at pH 6.5 (strong nitrite [NO_2_^−^] toxicity, strong interdependence) (Fig. 9). We found that the nitrite cross-feeding clones and the completely denitrifying clones again acquired genetic changes in genes annotated to cell motility (COG N) and signal transduction (COG T) (Fig. 9 and Additional file 1: Table S3). The nitrite cross-feeding clones and the completely denitrifying clones also acquired genetic changes in genes annotated to several additional functions, but these additional functions are largely limited to only a few clones (Fig. 9 and Additional file 1: Table S3). There are, however, a few notable exceptions. First, all of the completely denitrifying clones acquired non-synonymous point mutations in genes annotated to carbon metabolism (*pykA, fbp* or *gap-2*) while the nitrite cross-feeding clones did not (Additional file 1: Table S3). Second, all of the completely denitrifying clones acquired a point mutation in the intergenic region downstream of *nirS* (cytochrome cd1 nitrite reductase) and upstream of *nirT* (tetraheme cytochrome nirT), both of which are involved in nitrite reduction (assigned to the energy production and conversion category), while the nitrite cross-feeding clones did not (Additional file 1: Table S3). Finally, seven of the eight nitrite reducing clones acquired genetic changes in the *narX* or *narL* genes*,* both of which are components of a nitrogen oxide-sensing two-component regulatory system [35], while the cross-feeding clones did not (Additional file 1: Table S3). Taken together, our data indicate that nitrite cross-feeding can indeed affect the types of genetic changes that are acquired, and thus affect the trajectory of molecular evolution. This effect is greater when nitrite has strong toxic effects and promotes strong interdependence between the nitrite cross-feeding strains.

## Discussion

We found that, for our experimental system, substrate cross-feeding decreases the speed of molecular evolution at the strain level when nitrite has strong toxic effects (pH 6.5) (Fig. 3). One plausible explanation for this outcome is that the nitrite (NO_2_^−^) cross-feeding strains perform fewer metabolic processes, which would reduce the amount of genetic variation for selection to act upon. A second plausible explanation is that strong nitrite toxicity slows growth rates as we previously reported [12], which could also decrease the supply rate of new genetic changes, reduce the amount of genetic variation for selection to act upon, and slow the speed of molecular evolution [18, 19]. A final alternative explanation is that growth of one of the cross-feeding strains constrains the growth of the other, which would also reduce the amount of genetic variation for selection to act upon. For either explanation, the strains may start at a much lower fitness when nitrite toxicity is strong, thus increasing the number of available beneficial genetic changes. However, a subset of those beneficial genetic changes may only be available to the completely denitrifying strain. Indeed, we previously demonstrated that strong nitrite toxicity increases the number of available beneficial genetic changes [20], while other studies have also reported that low fitness genotypes evolve more rapidly than high fitness genotypes [36, 37].

Regardless of the exact underlying molecular cause of this observation, the important point here is that we observed no evidence that substrate cross-feeding increases the speed of molecular evolution at the strain level, which was our initial expectation. We initially reasoned that substrate cross-feeding would likely increase the speed of molecular evolution due to co-evolutionary processes, where synergistic or competitive interactions between different cross-feeding strains would increase the amount of genetic variation for selection to act upon, thus increasing the speed of molecular evolution. However, such co-evolutionary processes do not appear to be substantial determinants of the speed of molecular evolution for our experimental system, at least over the time-scales that we investigated in this study (700 generations of growth). It is plausible that co-evolutionary processes may become important over longer time-scales.

While substrate cross-feeding did not increase the speed of molecular evolution at the strain level (Figs. 2 and 3), substrate cross-feeding did increase the speed of molecular evolution at the community level (i.e., the sums of genetic changes acquired by the nitrite [NO_2_^−^] producing and reducing clones are greater than those for the completely denitrifying clones) (Figs. 4 and 5). The underlying cause of this is likely because nitrite cross-feeding enables the co-existence of two distinct microbial cell-types. Because the nitrite producing and reducing clones occupy distinct niche spaces and cannot displace each other in our experimental system, different genetic changes can accumulate in each strain, thus increasing the total number of genetic changes that accumulate within the total population.

While the effect described above is potentially weaker when nitrite (NO_2_^−^) toxicity is strong (Figs. 4 and 5), this is likely due to the fact that the completely denitrifying clones started at a much lower fitness than did the nitrite cross-feeding clones at the beginning of the evolution experiment [12]. Because the completely denitrifying clones have lower fitness, genetic changes may be available that have especially large beneficial effects to the completely denitrifying clones, which in turn may increase the speed of molecular evolution [20]. As an example, we previously found that the completely denitrifying clones accumulate more nitrite than do the cross-feeding clones [12]. We also found that genetic changes were available to the completely denitrifying clones that reduce the accumulation of nitrite, and thus had especially large beneficial effects [20]. Since these mutations are only available to the completely denitrifying clones, however, the completely denitrifying clones will have a faster speed of molecular evolution than the cross-feeding clones.

We also found that when nitrite (NO_2_^−^) has strong toxic effects, the completely denitrifying and nitrite cross-feeding clones acquired different types of genetic changes (Figs. 8, 9 and Additional file 1: Tables S2–S3). Specifically, the completely denitrifying clones always acquired genetic changes in genes annotated to carbon metabolism while the nitrite cross-feeding clones (and the nitrite reducing clones in particular) always acquired genetic changes in genes involved with denitrification. Thus, nitrite cross-feeding can indeed affect the trajectory of molecular evolution, where in our case the targets of selection switched from carbon utilization to nitrogen oxide metabolism depending on whether cells engage in nitrite cross-feeding or not.

## Conclusions

Our results demonstrate that substrate cross-feeding can affect both the speed and trajectory of molecular evolution, but these effects differ (and can even act in opposite directions) between the strain level and the community level. Moreover, the extent of these effects depends on the magnitude of toxicity of the cross-fed metabolite, and thus on the magnitude of interdependence between the substrate cross-feeding strains. Thus, substrate cross-feeding may indeed be an important interaction affecting the evolutionary processes and life histories of microorganisms.

## Methods

### Microbial strains and growth conditions

We used strains previously described elsewhere [12, 20, 25]. Briefly, we obtained wild-type *P. stutzeri* A1501 [21, 22] from the Biological Resource Center of Institut Pasteur (www.pasteur.fr/en/public-health/crbip) and used this strain to construct all of the mutant strains used in this study. We summarized all the genetic modifications here (Additional file 1: Table S1) and reported complete details of their construction elsewhere [12, 20, 25]. Briefly, the nitrite (NO_2_^−^) producing strain contains a loss-of-function mutation in the *nirS* gene to prevent nitrite reduction while the nitrite reducing strain contains a loss-of-function mutation in the *narG* gene to prevent nitrate (NO_3_^−^) reduction [12] (Fig. 1 and Additional file 1: Table S1). Additionally, all the strains contain a loss-of-function mutation in the *comA* gene to prevent the strains from internalizing extracellular DNA [12] (Additional file 1: Table S1). This reduced the probability that the nitrite producing and reducing strains would recombine with each other via transformation when grown together in co-culture during the evolution experiment. We constructed the mutant strains using derivatives of the pAW19 plasmid [38] as reported elsewhere [12].

To distinguish and quantify the relative frequencies of different strains when grown together in co-culture, we introduced DNA fragments containing the isopropyl-β-D-thiogalactopyranosid (IPTG)-inducible P_*lac*_ promoter located immediately upstream of the *egfp* or *echerry* gene into the nitrite (NO_2_^−^) producing and reducing strains [20, 25] (Fig. 1 and Additional file 1: Table S1). The *egfp* and *echerry* genes encode for green or red fluorescent proteins, respectively [39]. We introduced the DNA fragments using derivatives of the mini-Tn7T-LAC-Gm transposon and the pUC18T conditionally replicative delivery plasmid [40] as described in detail elsewhere [20, 25].

We cultivated all the *P. stutzeri* strains under aerobic conditions as described elsewhere [12, 20, 25]. Briefly, we cultivated the strains using a completely defined asparagine-citrate synthetic medium (ACS medium) [41] in 1-ml mixed batch reactors. We cultivated all the *P. stutzeri* strains under anaerobic conditions using nitrogen gas (N_2_)-sparged ACS medium amended with 10 mM of sodium nitrate (NaNO_3_) in 25-ml serum bottles fitted with gas-tight stoppers. We reported a complete description of the methods used to prepare and inoculate anaerobic ACS medium elsewhere [12]. We incubated all liquid cultures of *P. stutzeri* at 30 °C with shaking at 220 r.p.m.

### Experimental evolution

We experimentally evolved a total of eight nitrite (NO_2_^−^) cross-feeding co-cultures at each pH condition (pH 6.5 and 7.5) for a total of 16 co-cultures. Half of the co-cultures for each pH condition consisted of the nitrite producing strain carrying the *egfp* gene and the nitrite reducing strain carrying the *echerry* gene (Fig. 1). The other half of the co-cultures for each pH condition consisted of the nitrite producing strain carrying the *echerry* gene and the nitrite reducing strain carrying the *egfp* gene (Fig. 1). We used both combinations of *egfp* and *echerry*-encoding genes to periodically assess for cross-contamination between the co-cultures and to control for any potential differences in the metabolic costs associated with expressing the different fluorescent proteins. We did not add IPTG to the culture medium during the evolution experiment to avoid the metabolic costs of expressing the fluorescent proteins, and to therefore minimize the possibility of selecting for loss-of-function mutations in the *egfp* or *echerry* gene. We did not evolve the nitrite producing and reducing strains in isolation. Instead, we previously evolved the completely denitrifying strain in isolation [20], and we used the reported data as controls for this study.

We performed the evolution experiment using methodology described elsewhere [20]. Briefly, we streaked the nitrite (NO_2_^−^) producing or reducing strains separately onto lysogeny broth (LB) agar plates, inoculated one colony of each strain into a different test-tube containing 1 ml of aerobic ACS medium set to pH 7.5 or pH 6.5, and incubated the test-tubes for 24 h at 30 °C with continuous shaking. We then mixed the nitrite producing and reducing strains together into co-cultures at a 50:50 (vol:vol) ratio and diluted the co-cultures at a dilution of 1:25 (vol:vol) into serum bottles containing anaerobic ACS medium amended with 10 mM of sodium nitrate (NaNO_3_) to achieve a final volume of 20 ml. We next serially transferred the co-cultures in nitrogen gas (N_2_)-sparged ACS medium amended with 10 mM of sodium nitrate as the cell density-limiting substrate. We transferred each co-culture after entering stationary phase at a dilution of 1:200 (vol:vol) (with a few exceptions at the beginning of the evolution experiment when growth was slow and highly variable) for a total of approximately 700 generations of growth. Our estimate of 700 generations was based on the dilution, where we expect 7.64 generations from a 1:200 (vol:vol) dilution (i.e., 2^7.64^ = 200) [20]. This estimation is appropriate because we allowed the co-cultures to completely reduce all of the provided nitrogen oxides prior to each transfer [20].

At the same time, we performed an identical evolution experiment with the completely denitrifying strain and reported the outcomes in a previous study [20]. The experiment with the completely denitrifying strain also consisted of 16 cultures, half with the completely denitrifying strain carrying the *egfp* gene and the other half with the completely denitrifying strain carrying the *echerry* gene. We reported a complete description of the experimental and analytical methods for this experiment elsewhere [20].

We note here that there are differences in the fitness of the nitrite (NO_2_^−^) cross-feeding strains and the completely denitrifying strains depending on the toxicity of nitrite. At pH 7.5 (weak nitrite toxicity, weak interdependence), co-cultures of the nitrite cross-feeding strains collectively grow at approximately the same rate as the completely denitrifying strain [12]. At pH 6.5 (strong nitrite toxicity, strong interdependence), co-cultures of the nitrite cross-feeding strains collectively grow faster than the completely denitrifying strain [12]. This is because the nitrite cross-feeding strains accumulate less nitrite, and thus avoid its deleterious effects [12]. Finally, both co-cultures of the nitrite cross-feeding strains and the completely denitrifying strain grow slower at pH 6.5 (strong nitrite toxicity, strong interdependence) than at pH 7.5 (weak nitrite toxicity, weak interdependence). This could modify the supply rate of new genetic changes, and thus modify the amount of genetic variation for selection to act upon.

### Genome sequencing

We sequenced the genomes of evolved isolates using methodology described elsewhere [20]. Briefly, we streaked each evolved nitrite (NO_2_^−^) cross-feeding co-culture onto LB agar plates containing 10 μg ml^− 1^ of gentamicin and 0.1 mM of IPTG and picked a single colony of the nitrite producing and reducing strain (each colony expressed a different fluorescent protein) from each co-culture for genome sequencing. We grew the single clones in LB medium overnight and extracted the DNA with a Wizard Gemoic DNA purification kit (Promega, Madison, WI). We then sent the extracted DNA to the ETH Quantitative Genomics Facility (Basel, Switzerland) for sequencing. The genomes were sequenced with an Illumina HiSeq 200 sequencer (Illumina, San Diego, CA) with 100 cycles of paired-end sequencing. Primary data analysis, de-multiplexing and quality control analysis of the sequencing data were performed using FastQC (Illumina, San Diego, CA). We reported the complete set of parameters used for quality control elsewhere [17].

### Identification of genetic changes in the evolved clones

We analyzed the genome sequence data in collaboration with the ETH Genetic Diversity Centre (Zürich, Switzerland). We used PRINSEQ-lite v0.20.4 [42] to quality filter the raw reads, remove duplicate reads, and trim ambiguous base pairs. We used Breseq v.0.24rc5 [43] to identify genetic differences between each evolved genome and its ancestral reference genome. These genetic changes include synonymous and non-synonymous point mutations, insertions, deletions, and multiplications. We used the same procedures and parameters for sequence analysis as we used for the completely denitrifying strain in our previous study [20]. We reported the complete set of parameters used for sequence analysis elsewhere [17]. All genetic changes are summarized in Additional file 1: Tables S2–S3. All of the raw sequence reads are publically available in the European Nucleotide Archive (http://www.ebi.ac.uk/ena) under accession number PRJEB18464.

### Statistical analyses

We used non-parametric statistical tests for all data comparisons. We selected non-parametric statistical tests because the underlying distributions of our data were not initially known, and we had no a priori reason to assume the data would be normally distributed. Furthermore, we found that some of our data strongly deviated from a normal distribution [20], which invalidates the normality assumption typically required for parametric tests. We considered a *P*-value less than 0.05 to be statistically significant.

## Additional file

Additional file 1: **Table S1.** Nitrite (NO_2_^−^)-producing and reducing *Pseudomonas stutzeri* strains used in this study. **Table S2.** Genetic changes acquired by the nitrite (NO_2_^−^) producing or reducing clones (one clone each per culture) after 700 generations of evolution at pH 7.5 (weak nitrite toxicity, weak interdependence). **Table S3.** Genetic changes acquired by the nitrite (NO_2_^−^) producing or reducing clones (one clone each per culture) after 700 generations of evolution at pH 6.5 (strong nitrite toxicity, strong interdependence). (PDF 166 kb)

## Data Availability

All of the sequence reads analyzed for this publication are publically available in the European Nucleotide Archive (http://www.ebi.ac.uk/ena) under accession number PRJEB18464.

## Abbreviations

ACS: Asparagine-citrate synthetic medium
COG: Clusters of orthologous groups
ETH: Eidgenössische Technische Hochschule
GDC: Genetic diversity centre
IPTG: Isopropyl-β-D-thiogalactopyranosid
LB: Lysogeny broth
