# Supplementary material for: Substrate cross-feeding affects the speed and trajectory of molecular evolution within a synthetic microbial assemblage
Source: BMC Evol Biol. 2019 Jun 20;19:129. doi: 10.1186/s12862-019-1458-4 (PMC6584980; doi:10.1186/s12862-019-1458-4)
Supplement: Supplementary file 1 — Table S1. Nitrite (NO2−)-producing and reducing Pseudomonas stutzeri strains used in this study. Table S2. Genetic changes acquired by the nitrite (NO2−) producing or reducing clones (one clone each per culture) after 700 generations of evolution at pH 7.5 (weak nitrite toxicity, weak interdependence). Table S3. Genetic changes acquired by the nitrite (NO2−) producing or reducing clones (one clone each per culture) after 700 generations of evolution at pH 6.5 (strong nitrite toxicity, strong interdependence). (PDF 166 kb) [file 12862_2019_1458_MOESM1_ESM.pdf]

Additional file 1

Table S1 Nitrite (NO_2^-)-producing and reducing *Pseudomonas stutzeri* strains used in this study.

Strain	Relevant characteristics	Reference
**Producing strains**		
A1603gfp	*P. stutzeri* A1501 with Δ*comA*, Δ*nirS* and mini-Tn7T-LAC-Gm-*egfp*; Gm^R, $egfp^+$	23
A1603ech	*P. stutzeri* A1501 with Δ*comA*, Δ*nirS* and mini-Tn7T-LAC-Gm-*echerry*; Gm^R, $echerry^+$	23
**Reducing strains**		
A1602gfp	*P. stutzeri* A1501 with Δ*comA*, Δ*narG* and mini-Tn7T-LAC-Gm-*egfp*; Gm^R, $egfp^+$	23
A1602ech	*P. stutzeri* A1501 with Δ*comA*, Δ*narG* and mini-Tn7T-LAC-Gm-*echerry*; Gm^R, $echerry^+$	23

Table S2 Genetic changes acquired by the nitrite (NO_2^-) producing or nitrite reducing clones (one clone each per culture) after 700 generations of evolution at pH 7.5 (weak nitrite toxicity, weak interdependence).

Clone	Genome position	Genetic Change	Annotation[a]	Gene	Description
A1 Δ*nir*	2,796,851	C -> T	G176D (GGC -> GAC)	*flhA*	Flagellar biosynthesis protein
A1 Δ*nar*	2,771,093	A -> G	*505Q (TAG -> CAG)	*fleQ*	Transcriptional regulator
A1 Δ*nar*	3,552,142	C -> A	G176V (GGA -> GTA)	*PST_3282*	Fatty acid alpha hydroxylase
A2 Δ*nir*	733,863	C -> A	Intergenic (+902/-360)	*PST_0639 / PST_0640*	Type I restriction-modification system / phage Hau3 resistance protein
A2 Δ*nir*	1,486,000	RP3(+)Δ1bp	Coding (431-433/1317nt)	*flgE*	Flagellar hook protein
A2 Δ*nir*	1,809,904	C -> T	A86A (GCC -> GCT)	*PST_1684*	Hypothetical protein
A2 Δ*nir*	3,552,584	G -> T	Q29K (CAG -> AAG)	*PST_3282*	Fatty acid alpha hydroxylase
A2 Δ*nar*	2,063,012	G -> A	L144F (CTT -> TTT)	*PST_1911*	Hypothetical protein
A2 Δ*nar*	2,813,195	Δ4b	Coding (150-153/1758nt)	*fliF*	Flagellar MS-ring protein
A2 Δ*nar*	3,096,022	G -> A	L175L (CTG -> TTG)	*algI*	Alginate O-acetylation protein
A2 Δ*nar*	3,392,365	+TTT	Coding (934/1005nt)	*PST_3150*	Hypothetical protein
A3 Δ*nir*	3,392,355	+TGA	Coding (944/1005nt)	*PST_3150*	Hypothetical protein
A3 Δ*nar*	1,485,920	RP10(+)+3bp	Coding (353-355/1317nt)	*flgE*	Flagellar hook protein
A3 Δ*nar*	3,392,644	+CGA	Coding (833/1005)	*PST_3150*	Hypothetical protein
A4 Δ*nir*	443,079	6bp x 2	Duplication	*phoP*	Two-component response regulator
A4 Δ*nir*	2,808,475	G -> A	Q448* (CAA -> TAA)	*fliI*	Flagellum-specific ATP synthase
A4 Δ*nir*	3,036,324	G -> A	H199Y (CAC -> TAC	*aspS*	Aspartyl-tRNA synthetase
A4 Δ*nar*	442,808	T -> A	D97V (GAC -> GTC)	*phoP*	Two-component response regulator
A4 Δ*nar*	520,300	C -> G	R37P (CGC -> CCC)	*crc*	Catabolite repression control protein
A4 Δ*nar*	2,804,423	G -> A	Q254* (CAG -> TAG)	*fliK*	Flagellar hook-length control protein
B1 Δ*nir*	336,930	A -> G	E102G (GAG -> GGG)	*ompR*	Osmolarity response regulator
B1 Δ*nir*	2,532,546	C –> T	Noncoding (1207/2901nt)	*PST_2318*	23S ribosomal RNA
B1 Δ*nir*	2,771,721	Δ39bp	Coding (847-855/1515nt)	*fleQ*	Transcriptional regulator
B1 Δ*nir*	3,924,246	T -> A	L304Q (CTG -> CAG)	*PST_3634*	Thymidine phosphorylase
B1 Δ*nar*	2,804,555	C -> A	E210* (GAA -> TAA)	*fliK*	Flagellar hook-length control protein
B1 Δ*nar*	3,392,358	A -> C	I314S (ATC -> AGC)	*PST_3150*	Hypothetical protein
B2 Δ*nir*	1,199,102	C -> T	A265V (GCG -> GTG)	*lpxC*	UDP-3-O[3-hydroxymyristoyl]N-

					acetylglucosamine deacetylase
B2 *Δnir*	2,500,449	A -> G	G395G (GGT -> GGC)	*PST_2291*	Recombination factor protein RarA
B2 *Δnir*	2,808,847	C -> T	A324T (GCG -> ACG)	*fliI*	Flagellum-specific ATP synthase
B2 *Δnar*	2,809,371	G -> A	P149L (CCG -> CTG)	*fliI*	Flagellum-specific ATP synthase
B2 *Δnar*	3,392,466	C -> A	G278V (GGC -> GTC)	*PST_3150*	Hypothetical protein
B2 *Δnar*	4,005,451	G -> A	K219K (AAG -> AAA)	*PST_3703*	Hypothetical protein
B3 *Δnir*	2,799,641	7bp x 2	Duplication	*fliR*	Flagellar biosynthesis protein
B3 *Δnir*	3,332,325	35bp x 2	Duplication	*PST_3097 / PST_3098*	Hypothetical protein / cation-transporting P-type ATPase
B3 *Δnir*	3,392,466	C -> A	G278V (GGC -> GTC)	*PST_3150*	Hypothetical protein
B3 *Δnar*	1,198,532	C -> T	T75I (ACT -> ATT)	*lpxC*	UDP-3-O[3-hydroxymyristoyl]N-acetylglucosamine deacetylase
B3 *Δnar*	2,792,897	T -> C	Y69C (TAC -> TGC)	*fliA*	Flagellar biosynthesis sigma factor
B3 *Δnar*	4,057,685	Δ332bp	Intergenic (+1212/+764)	*PST_3751 / PST_3752*	CRISPR-associated Cas2 family protein / hypothetical protein
B4 *Δnir*	1,199,102	C -> T	A265V (GCG -> GTG)	*lpxC*	UDP-3-O[3-hydroxymyristoyl]N-acetylglucosamine deacetylase
B4 *Δnir*	1,486,023	RP3(-)Δ1bp	Coding (454-456/1317nt)	*flgE*	Flagellar hook protein
B4 *Δnar*	1,486,453	Δ8bp	Coding (886-893/1317nt)	*flgE*	Flagellar hook protein
B4 *Δnar*	3,107,691	C -> A	P359Q (CCG -> CAG)	*PST_2886*	Sensory box kinase/response regulator
B4 *Δnar*	3,392,466	C -> A	G278V (GGC -> GTC)	*PST_3150*	Hypothetical protein

Definitions: A *Δnir* clones; clones evolved from the ancestral strain 1602gfp; A *Δnar* clones; clones evolved from the ancestral strain 1603ech. B *Δnir* clones; clones evolved from the ancestral strain 1602ech. B *Δnar* clones; clones evolved from the ancestral strain 1603gfp. [a]The genomic position, type of amino acid change, and type of nucleotide change detected in each clone. Alphabetic letters indicate amino acid residues or nucleotides. Asterisks signify stop codons.

Table S3 Genetic changes acquired by the nitrite (NO_2^-) producing or nitrite reducing clones (one clone each per culture) after 700 generations of evolution at pH 6.5 (strong nitrite toxicity, strong interdependence).

**Clone**	**Genome position**	**Genetic Change**	**Annotation**[a]	**Gene**	**Description**
E1 *Δnir*	2,771,801	T -> C	M269V (ATG -> GTG)	*fleQ*	Transcriptional regulator
E1 *Δnir*	4,391,420	C -> T	R29W (CGG -> TGG)	*tctD*	Transcriptional regulatory protein
E1 *Δnir*	4,506,466	G -> A	Intergenic (+298/+10)	*pgm / adhA*	Phosphoglucomutase / alcohol dehydrogenase
E1 *Δnar*	2,075,529	A -> G	I90T (ATC -> ACC)	*acdA*	Acyl-CoA dehydrogenase
E1 *Δnar*	2,808,450	+G	Coding (1367/1380nt)	*fliI*	Flagellum-specific ATP synthase
E1 *Δnar*	3,818,976	G -> A	Intergenic (-54/+16)	*nirT / nirS*	Tetraheme protein NirT / cytochrome cd1 nitrite reductase
E1 *Δnar*	4,391,438	G -> T	A35S (GCC -> TCC)	*tctD*	Transcriptional regulatory protein
E2 *Δnir*	520,417	C -> G	Intergenic (-8/-78)	*crc / pyrE*	Catabolite repression control protein / orotate phosphoribosyltransf erase
E2 *Δnir*	2,795,867	A -> G	L504P (CTG -> CCG)	*flhA*	Flagellar biosynthesis protein
E2 *Δnar*	337,953	C -> G	P173A (CCG -> GCG)	*envZ*	Osmolarity sensor protein
E2 *Δnar*	993,754	T -> G	T209P (ACC -> CCC)	*narX*	Two-component sensor
E2 *Δnar*	1,485,633	Δ1::RP10(-)+3bp::+C	Coding (66-68(1317nt)	*flgE*	Flagellar hook protein
E2 *Δnar*	2,599,477	Δ6bp	Coding (997-1002/1293nt)	*PST_2380*	Porin
E2 *Δnar*	3,818,976	G -> A	Intergenic (-54/+16)	*nirT / nirS*	Tetraheme protein NirT / cytochrome cd1 nitrite reductase
E3 *Δnir*	1,108,945	Δ1bp	Coding (386/1446nt)	*PST_0997*	Sulfite reductase (NADPH) flavoprotein alpha-component
E3 *Δnir*	2,772,133	RP4(+)+8bp::Δ1bp	Coding (466-473/1515nt)	*fleQ*	Transcriptional regulator
E3 *Δnir*	3,355,746	G -> A	A20T (GCC -> ACC)	*PST_3117*	Hydroxypyruvate reductase
E3 *Δnir*	4,370,532	Δ15bp	Coding (270-284/513nt)	*PST_4028*	Long-chain acyl-CoA thioester hydrolase family protein
E3 *Δnar*	994,349	A -> G	N10N (AAT -> AAC)	*narX*	Two-component sensor
E3 *Δnar*	1,485,571	Δ1::RP10(-)+3bp::+C	Coding (4-6/1317nt)	*flgE*	Flagellar hook protein
E3 *Δnar*	3,818,977	C -> T	Intergenic (-55/+15)	*nirT / nirS*	Tetraheme protein NirT / cytochrome cd1

					nitrite reductase
E4 *Δnir*	248,848	A -> G	Intergenic (-129/-129	*PST_0212 / PST0213*	Hypothetical protein / hypothetical protein
E4 *Δnir*	1,108,945	Δ1bp	Coding (386/1446nt)	*PST_0997*	Sulfite reductase (NADPH) flavoprotein alpha-component
E4 *Δnir*	3,552,536	+G::RP10(+) +2bp	Coding (132-133/1251nt)	*PST_3282*	Fatty acid alpha hydroxylase
E4 *Δnir*	4,392,032	G -> A	A233T (GCC -> ACC)	*tctD*	Transcriptional regulatory protein
E4 *Δnar*	992,029	Δ1bp	Coding (396/609nt)	*narL*	Two-component response regulator
E4 *Δnar*	2,808,450	+G	Coding (1367/1380nt)	*fliI*	Flagellum-specific ATP synthase
E4 *Δnar*	3,818,976	G -> A	Intergenic (-54/+16)	*nirT / nirS*	Tetraheme protein NirT / cytochrome cd1 nitrite reductase
E4 *Δnar*	4,231,226	T -> C	I18V (ATT -> GTT)	*PST_3901*	Hypothetical protein
F1 *Δnir*	2,097,947	C -> T	A484T (GCG -> ACG)	*PST_1939*	Molybdopterin oxidoreductase
F1 *Δnir*	2,772,023	T -> C	K195E (AAG -> GAG)	*fleQ*	Transcriptional regulator
F1 *Δnir*	4,392,032	G -> A	A233T (GCC -> ACC)	*tctD*	Transcriptional regulators protein
F1 *Δnar*	992,035	Δ1bp	Coding (390/609nt)	*narL*	Two-component response regulator
F1 *Δnar*	1,695,585	T -> C	E182G (GAA -> GGA)	*PST_1582*	Hypothetical protein
F1 *Δnar*	2,289,691	6bp x 2	Duplication	*PST_2110*	Methyl-accepting chemotaxis protein
F1 *Δnar*	2,801,806	G -> A	R148* (CGA -> TGA9	*fliN*	Flagellar motor switch protein
F1 *Δnar*	3,818,977	C -> T	Intergenic (-55/+15)	*nirT / nirS*	Tetraheme protein nirT / cytochrome cd1 nitrite reductase
F1 *Δnar*	4,085,150	Δ266bp	Intergenic (+1677/+365)	*PST_3751 / PST_3752*	CRISPR-associated Cas2 family protein / hypothetical protein
F2 *Δnir*	514,278	C -> T	R678H (CGC -> CAC)	*spoT*	Guanosine-3,5-bis(diphosphate)3-pyrophosphohydrolas e
F2 *Δnir*	2,800,913	Δ12bp	Coding (145-156/600nt)	*fliP*	Flagellar biosynthetic protein
F2 *Δnar*	992,006	+AG	Coding (419/609)	*narL*	Two-component response regulator
F2 *Δnar*	1,485,918	Δ1::RP10(-)+4bp	Coding (351-354/1317nt)	*flgE*	Flagellar hook protein
F2 *Δnar*	3,552,604	G -> T	P22H (CCC -> CAC)	*PST_3282*	Fatty acid alpha hydroxylase
F2 *Δnar*	3,818,976	G -> A	Intergenic (-54/+16)	*nirT / nirS*	Tetraheme protein nirT / cytochrome cd1 nitrite reductase
F3 *Δnir*	1,339,349	Δ1bp	Coding (612/627nt)	*rnc*	Ribonuclease III
F3 *Δnar*	743,600	Δ1bp::RP10(-)+3bp::+C	Coding (1455-1457/1977nt)	*PST_0647*	Type I restriction-modification system, M subunit

F3 *Δnar*	991,916	C -> T	G170D (GGC -> GAC)	*narL*	Two-component response regulator
F3 *Δnar*	2,771,884	G -> A	A241V (GCG -> GTG)	*fleQ*	Transcriptional regulator
F3 *Δnar*	3,818,976	G -> T	Intergenic (-54/+16)	*nirT / nirS*	Tetraheme protein nirT / cytochrome cd1 nitrite reductase
F4 *Δnir*	2,812,145	9bp x 2	Duplication	*fliF*	Flagellar MS-ring protein
F4 *Δnir*	2,840,586	C -> T	G34D (GGC -> GAC)	*fabF*	3-oxoacyl-(acyl carrier protein)
F4 *Δnir*	2,859,528	T -> C	W158R (TGG -> CGG)	*PST_2640*	Hypothetical protein
F4 *Δnir*	4,079,990	C -> T	R256R (CGC -> CGT)	*holA*	DNA polymerase III subunit delta
F4 *Δnar*	993,102	G -> A	A426V (GCG -> GTG)	*narX*	Two-component sensor
F4 *Δnar*	3,818,976	G -> A	Intergenic (-54/+16	*nirT / nirS*	Tetraheme protein nirT / cytochrome cd1 nitrite reductase

Definitions: E *Δnir* clones; clones evolved from the ancestral strain 1602gfp; E *Δnar* clones; clones evolved from the ancestral strain 1603ech. F *Δnir* clones; clones evolved from the ancestral strain 1602ech. F *Δnar* clones; clones evolved from the ancestral strain 1603gfp. [a]The genomic position, type of amino acid change, and type of nucleotide change detected in each clone. Alphabetic letters indicate amino acid residues or nucleotides. Asterisks signify stop codons.
